# Treatment escalation patterns to start biologics in refractory moderate juvenile dermatomyositis among members of the Childhood Arthritis and Rheumatology Research Alliance

**DOI:** 10.1186/s12969-022-00785-5

**Published:** 2023-01-06

**Authors:** Matthew A. Sherman, Hanna Kim, Katelyn Banschbach, Amanda Brown, Harry L. Gewanter, Bianca Lang, Megan Perron, Angela Byun Robinson, Jacob Spitznagle, Cory Stingl, Grant Syverson, Heather O. Tory, Charles H. Spencer, Stacey E. Tarvin

**Affiliations:** 1 Muscle Disease Unit, Laboratory of Muscle Stem Cells and Gene Regulation, National Institute of Arthritis and Musculoskeletal and Skin Diseases, National Institutes of Health, 9000 Rockville Pike 50 South Drive Building 50, Room 1142, 20892 Bethesda, MD USA; 2 Division of Rheumatology, Children’s National Hospital, Washington, DC USA; 3 Juvenile Myositis Pathogenesis and Therapeutics Unit, National Institute of Arthritis Musculoskeletal and Skin Diseases, National Institutes of Health, Bethesda, MD USA; 4 Division of Rheumatology, Department of Pediatrics, University of Washington/Seattle Children’s Hospital, Seattle, WA USA; 5 University of Arkansas for Medical Sciences and Arkansas Children’s Hospital, Little Rock, AR USA; 6 Children’s Hospital of Richmond at VCU, Richmond, VA USA; 7 IWK Health, Dalhousie University, Halifax, NS Canada; 8 Department of Pediatric Rheumatology, Children’s Hospital Colorado, Aurora, CO USA; 9 Pediatric Rheumatology, Cleveland Clinic Children’s Hospital, Cleveland, OH USA; 10 Division of Rheumatology, Department of Pediatrics, University of Washington/Seattle Children’s Hospital, Seattle, WA USA; 11 Department of Pediatrics, Spectrum Health, Grand Rapids, MI USA; 12 Sanford Health, Fargo, ND USA; 13 Connecticut Children’s Medical Center, Hartford, CT, USA and University of Connecticut School of Medicine, Farmington, Farmington, CT USA; 14 University of Mississippi Medical Center, Batson Children’s Hospital, Jackson, MS USA; 15 Division of Rheumatology, Department of Pediatrics, Indiana University School of Medicine, Indianapolis, IN USA

**Keywords:** Juvenile dermatomyositis, Dmards, Biologics

## Abstract

**Background:**

Despite new and better treatments for juvenile dermatomyositis (JDM), not all patients with moderate severity disease respond adequately to first-line therapy. Those with refractory disease remain at higher risk for disease and glucocorticoid-related complications. Biologic disease-modifying antirheumatic drugs (DMARDs) have become part of the arsenal of treatments for JDM. However, prospective comparative studies of commonly used biologics are lacking.

**Methods:**

The Childhood Arthritis and Rheumatology Research Alliance (CARRA) JDM biologics workgroup met in 2019 and produced a survey assessing current treatment escalation practices for JDM, including preferences regarding use of biologic treatments. The cases and questions were developed using a consensus framework, requiring 80% agreement for consensus. The survey was completed online in 2020 by CARRA members interested in JDM. Survey results were analyzed among all respondents and according to years of experience. Chi-square or Fisher’s exact test was used to compare the distribution of responses to each survey question.

**Results:**

One hundred twenty-one CARRA members responded to the survey (denominators vary for each question). Of the respondents, 88% were pediatric rheumatologists, 85% practiced in the United States, and 43% had over 10 years of experience. For a patient with moderately severe JDM refractory to methotrexate, glucocorticoids, and IVIG, approximately 80% of respondents indicated that they would initiate a biologic after failing 1–2 non-biologic DMARDs. Trials of methotrexate and mycophenolate were considered necessary by 96% and 60% of respondents, respectively, before initiating a biologic. By weighed average, rituximab was the preferred biologic over abatacept, tocilizumab, and infliximab. Over 50% of respondents would start a biologic by 4 months from diagnosis for patients with refractory moderately severe JDM. There were no notable differences in treatment practices between respondents by years of experience.

**Conclusion:**

Most respondents favored starting a biologic earlier in disease course after trialing up to two conventional DMARDs, specifically including methotrexate. There was a clear preference for rituximab. However, there remains a dearth of prospective data comparing biologics in refractory JDM. These findings underscore the need for biologic consensus treatment plans (CTPs) for refractory JDM, which will ultimately facilitate comparative effectiveness studies and inform treatment practices.

**Supplementary Information:**

The online version contains supplementary material available at 10.1186/s12969-022-00785-5.

## Background

The idiopathic inflammatory myopathies (IIM) are a heterogenous group of autoimmune connective tissue diseases characterized by chronic skeletal muscle inflammation. There are different clinical myositis subtypes. Juvenile dermatomyositis (JDM), which includes typical skin findings such as Gottron’s papules or heliotrope rash, is the most common clinical subtype in childhood. Myositis-specific autoantibodies (MSAs), which typically define distinct clinical phenotypes, provide a further layer of classification. Different clinicoserologic subgroups are associated with different disease manifestations and outcomes [1, 2].

Patients with moderately severe JDM that is refractory to first-line therapies, mainly systemic glucocorticoids and a conventional disease modifying anti-rheumatic drug (DMARD), in particular methotrexate (MTX), remain difficult to treat. Moreover, patients with ongoing disease activity are at higher risk for complications such as calcinosis and lipodystrophy as well as sequelae related to chronic glucocorticoid use [3, 4]. Since the results of the 2013 Rituximab in Myositis (RIM) trial, which demonstrated effectiveness of rituximab as a treatment for refractory myositis despite not meeting the primary endpoint of the study, biologic DMARDs have been adopted widely, both in the United States and abroad [5–9]. A growing literature of case reports of patients with refractory JDM has suggested the effectiveness of multiple biologics, including abatacept, tocilizumab, and tumor necrosis factor (TNF) inhibitors in addition to rituximab [4, 10, 11]. However, prospective comparative studies of commonly used biologics in patients with JDM are lacking.

In anticipation of developing Consensus Treatment Plans (CTPs) for the use of biologics in refractory moderately severe JDM, the biologics subcommittee of the Childhood Arthritis and Rheumatology Research Alliance (CARRA) JDM workgroup produced a survey to assess current treatment escalation practices for JDM, including preferences for when and which biologics are used. The eventual goal of the aforementioned CTPs is to facilitate prospective comparative effectiveness studies that will inform and improve treatment of refractory JDM.

## Methods

The biologics CTP committee of the CARRA JDM workgroup met in Louisville, Kentucky on 13 April 2019 at the annual CARRA meeting. The group met to discuss developing the biologic CTPs and to address questions that remained regarding implementation and acceptance of the biologic treatment options by CARRA members. A group of 20 JDM biologic CTP committee members including pediatric rheumatologists, fellows-in-training, researchers, and parents of children with JDM divided into four self-selected subgroups to devise case scenarios addressing the outstanding questions. Two facilitators (SET and CHS) discussed the background and objectives of the CTPs as well as the consensus methodology and plan for developing the cases. Additionally, it is important to note that this project was devised prior to the emerging literature regarding the role of janus kinase (JAK) inhibition in JDM.

The cases were designed to address four topics: (1) Which non-biologic DMARDs should be trialed, and for how long, prior to starting a biologic; (2) Should patients enrolled in one of the CTPs be biologically naïve; if not, how long after exposure should they be permitted to enroll; (3) Should glucocorticoids for bridging therapy be permitted upon starting a biologic and what does “bridging” mean; and (4) What should be the role of IVIG in the CTPs.

Within each subgroup, input was sought from all attendees to develop the cases. The cases were then presented to the entire group for feedback and discussion. A benchmark of 80% agreement for each question was required to achieve consensus. When there was not consensus, revisions were suggested and voted on by a show of hands until 80% consensus was achieved. After the meeting, the cases were further refined over email by each of the four groups and then submitted to the facilitators. These cases were developed into an online survey (Supplemental document 1), which was distributed in August 2020 to 188 voting and trainee physician CARRA members who expressed interest in caring for patients with JDM. Respondents completed the survey over August-September 2020. Demographics including specialty, location of practice, and years of experience were later captured.

The biologic CTP survey and development process were approved by the Indiana University IRB.

Survey results were analyzed among the respondents at large as well as stratified by years of experience. Chi-square or Fisher’s exact test, where appropriate, was used to compare the distribution of responses to each survey question. R Statistical Software (Version 4.1.0; R Core Team 2021) was used to perform the analyses [12]. Significance was set at *p* < 0.05.

## Results

### Respondents

There were 127 initial responses, including 3 who opted out from the survey and 3 who had replied twice. Overall, there were 121 responses (64% response rate) and demographic information was available for 99 (Table 1). The majority (88%) identified as pediatric rheumatologists, and most of the others (9%) were combined medicine-pediatric rheumatologists. The majority of respondents practiced in North America, with 85% in the United States and 7% in Canada. Over 40% had been in practice for more than 10 years.

**Table 1:** Respondent characteristics

Characteristic	*N* = 99^*1*^
Specialty	
Pediatric rheumatology	87 (88%)
Med-Peds rheumatology	9 (9%)
Pediatric rheumatology and Allergy/Immunology	1 (1%)
Dermatology	1 (1%)
Other	1 (1%)
Location of practice	
United States	84 (85%)
Canada	7 (7%)
United Kingdom	2 (2%)
Middle East	5 (5%)
India	1 (1%)
Years of experience	
< 1	10 (10%)
1–5	24 (24%)
6–10	22 (22%)
> 10	42 (43%)
Unknown	1
^*1*^ n (%)	

Demographic features available of survey respondents

### Case escalation patterns

#### 8 Weeks after initiation of treatment

After 8 weeks of no response to treatment in a patient with moderately severe JDM who had been managed according to Protocol B (Supplemental document 1) per the 2010 CARRA consensus treatment protocols for moderately severe JDM [13], most respondents (*n* = 79, 71%) indicated that they would add an agent to MTX rather than switch from MTX (*n* = 14, 12%) or continue without change (*n* = 10, 9%). Slightly more respondents preferred to add a biologic (*n* = 41, 48%) than a non-biologic DMARD (*n* = 36, 42%). Mycophenolate (MMF) was the most preferred non-biologic DMARD addition (*n* = 22, 63%) followed by a calcineurin inhibitor (CNI) (*n* = 7, 20%); azathioprine (AZA) and hydroxychloroquine were selected less frequently (*n* = 3, 9% for each). Among those who would switch from MTX, most opted for a non-biologic DMARD (*n* = 12, 80%) and all but one selected MMF (*n* = 12, 92%).

#### 12 weeks after initiation of treatment

If the clinical course was unchanged after 12 weeks in a patient with moderately severe JDM on conventional treatments per the 2010 CARRA consensus treatment protocols, most of those surveyed (*n* = 61, 55%) preferred adding another agent instead of switching from MTX (*n* = 28, 25%) or continuing without change (*n* = 11, 10%). A biologic was the preferred addition (*n* = 51, 80%) at this point compared to a non-biologic DMARD (*n* = 12, 19%). MMF was again preferred among those who would add a non-biologic DMARD (*n* = 7, 58%) as well as those who would switch from methotrexate to a different non-biologic DMARD (*n* = 12, 55%); a CNI was the second preference in each situation.

#### 16 weeks after initiation of treatment

By 16 weeks without improvement in a patient with moderately severe JDM on conventional treatments per the 2010 CARRA consensus treatment protocols, over half responded that they would start a biologic (*n* = 56, 54%). Equal numbers of respondents (*n* = 17, 16%) indicated that they would wait longer, either 20 weeks or 24 weeks. Seven others (7%) specified that they would have started a biologic sooner, ranging between 6 and 16 weeks after initiation of treatment.

#### DMARD thresholds to start a biologic

**Fig. 1:**
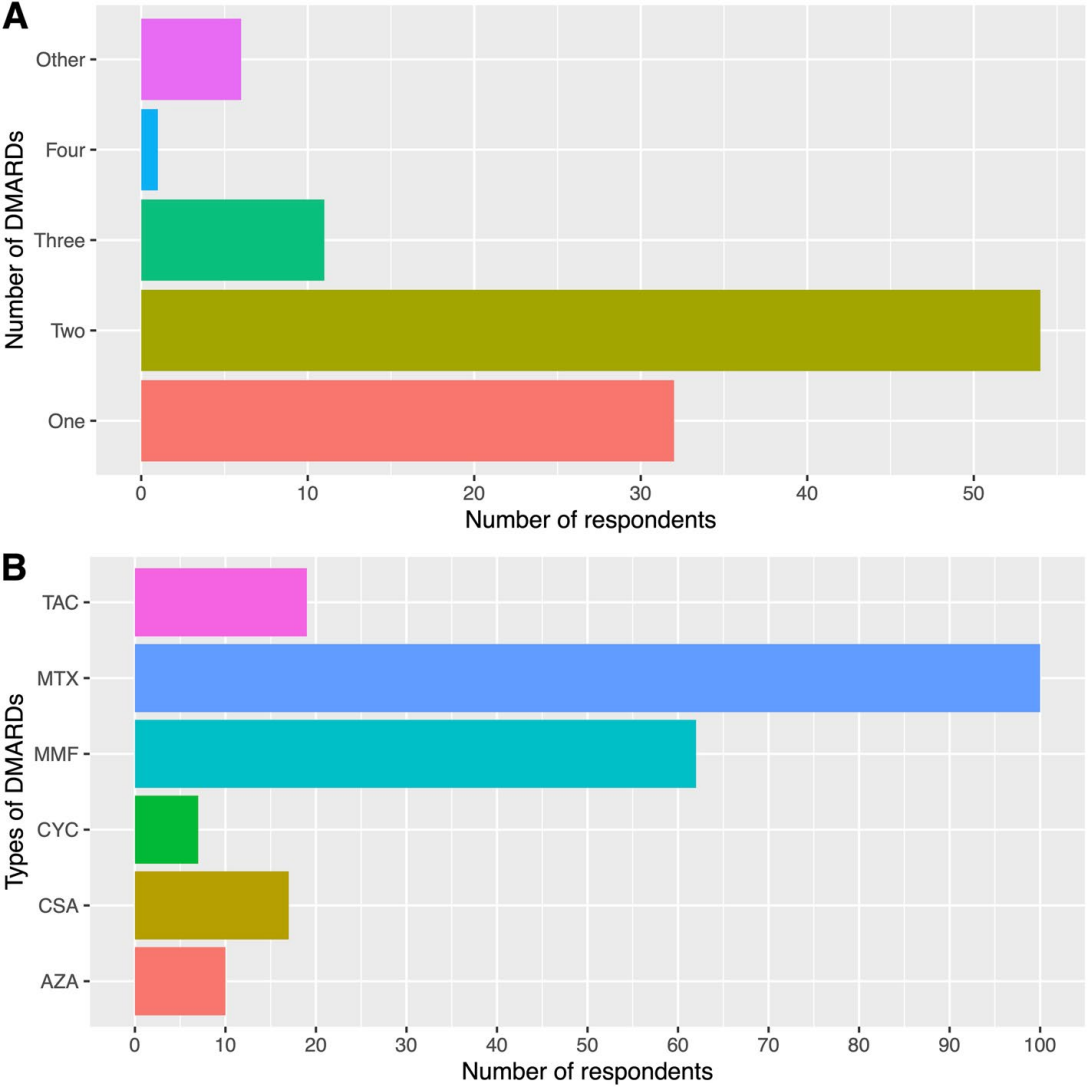
Disease modifying anti-rheumatic drug (DMARD) escalation patterns. Respondents were asked to select the number (**A**) and types (**B**) of conventional DMARDs that a patient should fail prior to starting a biologic. *Abbreviations* *AZA* azathioprine, *CSA* cyclosporine, *CYC* cyclophosphamide, *MMF* mycophenolate, *MTX* methotrexate, *TAC * tacrolimus

There were also different practice patterns with regard to the number and type of non-biologic DMARDs that respondents thought patients should have tried prior to initiating a biologic (Fig. 1). The majority (*n* = 86, 83%) would start a biologic after failing one (*n* = 32, 31%) or two (*n* = 54, 52%) non-biologic DMARDs. Few (*n* = 11, 11%) would require a patient to try and fail three non-biologic DMARDs, and two respondents (1.9%) considered 1 DMARD in combination with intravenous immunoglobulin (IVIG) a sufficient trial. Nearly all of the respondents (*n* = 100, 96%) indicated that methotrexate should be one of the non-biologic DMARDs trialed. MMF (*n* = 62, 60%) and a CNI (*n* = 36, 35%) were other common selections.

### Biologic practices

#### Choice of biologic agent

**Fig. 2:**
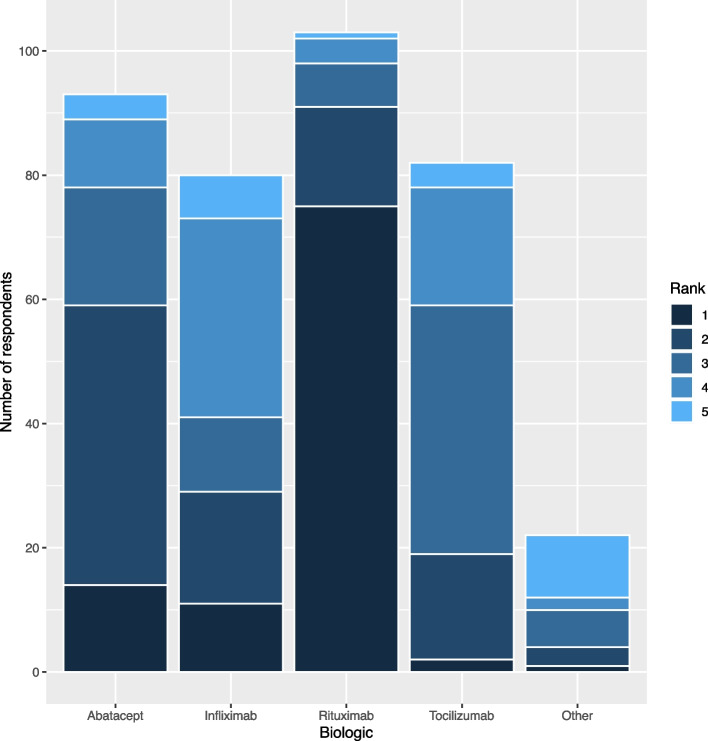
Ranking of biologics. Respondents were asked to rank the above biologic therapies in order of preference from 1 (first preference) to 5 (last preference). By weighted average, respondents favored rituximab (1.45) followed by abatacept (2.42), tocilizumab (3.07), infliximab (3.08), and other (3.77)

In response to the above case, respondents were asked to rank their preferred biologic out of a list of five including rituximab, abatacept, tocilizumab, infliximab, or other (Fig. 2). By weighted average rank, with one indicating top preference, there was a clear preference for rituximab as the first choice (1.45) followed by abatacept (2.42). Tocilizumab (3.07) and infliximab (3.08) were ranked nearly equally, and “other” was ranked last (3.77). Of those who specified “other” as a fifth choice, 10 indicated that they would consider a JAK inhibitor, either tofacitinib or baricitinib.

#### History of biologic exposure

Prior biologic exposure was queried as an exclusionary factor for future JDM biologic CTPs. Respondents were evenly split (50% in favor, 50% against) as to whether patients who were previously treated with a biologic should be included. Those in favor were asked additional questions regarding prior biologic exposure. Regarding the potential interval before enrollment, a washout of 4–5 half-lives or 1 month was preferred (*n* = 36, 72%) for biologics other than rituximab compared to enrollment without restriction (*n* = 13, 26%). For rituximab, most respondents would include patients 6 months after the last dose with detectable B cells (*n* = 19, 38%) compared with over 6 months (*n* = 12, 24%) or no restriction (*n* = 9, 18%). For those who specified other, 4 would include patients 3–4 months after the initial dose of rituximab and 4 would include patients whenever B cells became detectable.

Another consideration was whether to permit patients, following a wait period between cessation of the biologic and participation in a CTP, to enroll in the treatment arm with the same biologic with which they were previously treated. If a patient had failed a TNF inhibitor, most of those surveyed would allow them to enroll in this arm (76%). However, of these respondents 54% stipulated that the patient would have to try a different TNF inhibitor. In the case of prior benefit from a TNF inhibitor, most would also allow them to enroll in this arm (83%), and among them more would permit this regardless of the previous TNF inhibitor (61%). For abatacept, tocilizumab, and rituximab, there was agreement that if they had failed one of those specific biologics they should be excluded from enrollment (68%, 69%, and 63%, respectively) but if they had past clinical benefit they should be allowed to enroll (76%, 80%, and 80%, respectively). Additionally, if a patient had just started treatment with an included CTP treatment arm most of those surveyed (82%) would not exclude them from participation in CTP enrollment. Over half (55%) of respondents would permit enrollment up to 1 month after starting the biologic. Some would even permit enrollment up to 2–6 months after, if the treatment was consistent with the CTP and appropriate documentation was available.

### Steroid practices

Respondents were queried for their practices with glucocorticoids in the setting of disease flare prompting initiation of a biologic. In the case of a patient flaring while on monthly pulse steroids (IV methylprednisolone 30 mg/kg, max dose 1000 mg) and daily oral steroids, many of those surveyed would consider escalating pulse methylprednisolone regimen (*n* = 47, 47%). Specifically, approximately equal numbers would consider weekly pulses for 4 weeks (*n* = 16, 16%), daily pulses for 3 consecutive days (*n* = 15, 15%), or either of these options (*n* = 16, 16%). Around one quarter of respondents (*n* = 23, 24%) would continue the current regimen and 17 (18%) would entertain either no change or either of the pulse options.

In the case of a patient flaring while off steroids, there was consensus to resume steroids (*n* = 79, 81%). If steroids were to be resumed, most respondents selected some regimen of pulse methylprednisolone (*n* = 53, 57%). Specifically, most preferred multiple pulse doses (*n* = 44, 47%) with 17 (18%) preferring pulses for 3 consecutive days, 13 (14%) for weekly pulses for 4 weeks, and 14 (15%) for either option; only 9 (10%) opted for a single pulse dose. Over one quarter (*n* = 26, 28%) would start only oral steroids and another 7 (8%) would start oral in addition to pulse steroids. For ongoing treatment, most preferred daily oral steroids with a taper (*n* = 57, 63%) over the combination of daily steroids with a taper and monthly pulse methylprednisolone (*n* = 34, 37%).

### IVIG practices

Respondents were also asked whether they would continue IVIG in the case of persistent disease prompting initiation of a biologic. There was consensus to continue IVIG in this situation (*n* = 85, 88%) but not with regard to the duration. Approximately as many respondents would continue IVIG until reaching definition of clinical improvement as would continue at their discretion (*n* = 40, 47% v *n* = 41, 48%).

### Differences in treatment practices by years of experience

A stratified analysis of survey responses based on years of experience was performed among those respondents who had full demographic information available. Of those 98 respondents, 53 had fewer than 10 years and 45 had at least 10 years of experience. Treatment practices varied modestly between the two groups. There was a significant difference in the distribution of responses regarding at which time point they would start a biologic following diagnosis in case 1 (*p* = 0.046). At least half of each group indicated that they would do so at 16 weeks after diagnosis (63% and 50% for those with fewer than 10 years and those with at least 10 years of experience, respectively). An additional five respondents with at least 10 years of experience specified that they would have started a biologic before 16 weeks.

The only other difference between the groups was in the distribution of responses regarding the time up to which a patient who started a biologic should be permitted to enroll in a CTP (*p* = 0.028), assuming it was consistent with a theoretical biologic CTP. Most (79%) respondents with fewer than 10 years of experience responded 1 month compared to 35% of those with at least 10 years of experience; an additional three within this latter group indicated that they would permit patients to enroll after a long period, up to 3–6 months, if there was appropriate documentation.

## Discussion

Refractory JDM remains challenging to treat, and these patients are at higher risk of disease and glucocorticoid-related complications [3, 4]. The dearth of comparative studies for second-line treatments, in particular biologic DMARDs, compounds this difficulty. In the present survey, treatment practices of a large sample of pediatric rheumatology providers predominantly in North America are described.

Thresholds by which respondents would escalate treatment to a biologic were apparent. Approximately 55% of respondents to question 12 indicated that they would add a biologic to the regimen for the patient presented in case 1 (Supplemental document 1) if there was no improvement by 4 months from diagnosis, and, moreover, there was a growing preference for biologics as early as 8 weeks among those who would add to this regimen. Over 80% of respondents would begin a biologic in the case after the patient failed 1–2 conventional DMARDs, of which MTX followed by MMF were the preferred agents. These preferences are similar to the treatment recommendations for juvenile dermatomyositis patients with mild/moderate or refractory disease outlined in the 2017 Single Hub and Access point for pediatric Rheumatology in Europe (SHARE) guidelines for juvenile dermatomyositis [14]. The SHARE guidelines suggest escalating treatment, including with either conventional or biologic DMARDs, within the first 3 months if no improvement. However, in contrast to the case in the present survey, which included systemic glucocorticoids, methotrexate, and IVIG at onset, the SHARE recommendations for initial management include systemic glucocorticoids and methotrexate, whereas IVIG is suggested for intensifying treatment in refractory disease.

MTX remains a preferred first-line treatment for moderate JDM, which may reflect dissemination of the 2010 CARRA CTP for moderately severe JDM and the 2017 SHARE guidelines [13, 14]. In refractory cases prior to 16 weeks, respondents who preferred to add another conventional DMARD favored MMF over a CNI. These results resemble the responses to a similar case of refractory moderate JDM considered in a 2018 survey of pediatric rheumatology and neurology providers in Germany [8]. There, after six weeks 73% preferred adding an additional treatment to a foundation of glucocorticoids, methotrexate, and hydroxychloroquine. IVIG and intravenous methylprednisolone (IVMP) were the favored agents of those who would add or change treatments, whereas cyclosporin and MMF were less popular and even fewer respondents opted for rituximab, TNF inhibition, or tocilizumab. However, it is important to note that both IVIG and IVMP were already part of the treatment regimen for the case included in the present survey.

A recent international survey investigating provider perspectives regarding the utility of myositis autoantibodies in patients with IIM found that they are considered to be an important component of clinical practice [15]. Similarly, although not specifically queried in the survey, respondents in their comments emphasized the role of these autoantibodies in determining appropriate treatment regimens. Some respondents expressed that autoantibody status was an indication not just for early escalation but also as a guide for selecting a biologic. Specifically, rituximab was the agent of choice for and would be started sooner in myositis autoantibody-positive patients with refractory JDM. This preference for rituximab in patients with myositis autoantibodies, similar to the rank of rituximab first by the respondents at large, may be based on the efficacy of this biologic in refractory JDM demonstrated by RIM trial [5]. A proclivity for rituximab among biologics in moderate refractory JDM was also found in the German survey [8]. There, other biologics considered in order of preference were TNF inhibition, tocilizumab, and abatacept, whereas here abatacept was ranked second by weighted average followed by approximately equal preference for infliximab and tocilizumab.

JAK inhibition was proposed by several of the respondents who ranked an alternative agent to the four specified biologics. These small molecule drugs inhibit cell signaling pathways, including interferon among others, and have an emerging role in the treatment of IIM. JAK inhibitors have been reported to be effective in case series of juvenile IIM including those with anti-MDA5 autoantibodies [16–20]. Though promising, new concerns regarding safety and adverse events, specifically related to potential cardiovascular effects and malignancies in adults, require further investigation [21].

In some areas of JDM care, there are differences in treatment practices based on provider experience. A 2017 CARRA survey investigating management of JDM associated calcinosis revealed that providers who had taken care of more than 10 cases, compared to those with less experience, more frequently used certain immunomodulatory medications such as colchicine as well as alternative treatments such as bisphosphonates [22]. An earlier CARRA survey, however, was notable for marked treatment variability among providers, but there was no statistically significant difference in the use of first line treatments based on the number of new JDM patients a provider had seen over the last five years [23]. In this survey, there also were no clinically relevant statistically significant differences between respondents stratified by whether they had at least 10 or fewer than 10 years of experience.

Finally, respondents were divided regarding whether to include patients in a prospective CTP if they had previous exposure to a biologic; among those in favor, the overall preference was to enroll patients only after a washout period. Glucocorticoids remain key to addressing disease flare. Respondents in general would resume or escalate steroids when initiating a biologic, but variation in treatment regimens remain and may be in part driven by provider practice and heterogeneity in disease features and severity. IVIG was also considered to be an important part of flare management and there was consensus that it should be continued upon starting a biologic.

This project has several limitations. Although the cases presented in the survey were modeled on real patients, they were hypothetical; care in a real-world setting may differ. The respondents to this survey are CARRA members who had expressed an interest in juvenile myositis and therefore may not be representative of practices among pediatric rheumatology providers more broadly. Due to incomplete demographic information, all of the respondents were not included in the stratified analysis. However, strengths of this survey include the focus on refractory moderately severe JDM, relatively large total sample size, and representation of practice patterns from pediatric rheumatology providers throughout the continental United States.

## Conclusion

This large survey querying approaches to refractory moderate JDM among CARRA members with an interest in JDM revealed wide variability in treatment practices. However, respondents overall preferred to initiate a biologic DMARD earlier in disease course after trying up to two conventional DMARDs. Though rituximab was favored among biologic agents, there is insufficient evidence from prospective comparative studies to determine the optimal treatment for refractory JDM. Given the apparent widespread use of biologic DMARDs, these findings warrant the development of biologic CTPs for refractory JDM to determine the relative effectiveness of these treatments, the ultimate goals of which are to inform treatment practices and to improve patient outcomes.

## Supplementary information

**Additional file 1.**

## Data Availability

The datasets used and/or analyzed for the current study are available from the corresponding author upon reasonable request.

## Abbreviations

AZA: azathioprine
CARRA: Childhood Arthritis and Rheumatology Research Alliance
CNI: calcineurin inhibitor
CSA: cyclosporine
CTP: consensus treatment plan
DMARD: disease modifying anti-rheumatic drug
IIM: idiopathic inflammatory myopathies
IVIG: intravenous immunoglobulin
IVMP: intravenous methylprednisolone
JAK: janus kinase
JDM: juvenile dermatomyositis
MTX: methotrexate
MMF: mycophenolate
MSA: myositis-specific autoantibody
RIM: rituximab in myositis trial
SHARE: Single Hub and Access point for pediatric Rheumatology in Europe
TAC: tacrolimus
TNF: tumor necrosis factor
