# Supplementary material for: Treatment escalation patterns to start biologics in refractory moderate juvenile dermatomyositis among members of the Childhood Arthritis and Rheumatology Research Alliance
Source: Pediatr Rheumatol Online J. 2023 Jan 6;21:3. doi: 10.1186/s12969-022-00785-5 (PMC9825021; doi:10.1186/s12969-022-00785-5)
Supplement: Supplementary file 1 — Additional file 1. [file 12969_2022_785_MOESM1_ESM.pdf]

1. Please indicate if you would like to participate in the survey.
    a. Yes, I would like to participate.
    b. No, I would like to opt out.
    c. No, I would like to opt out AND would like to be removed from the JDM listserv.
2. Andie is an 8yo female with newly diagnosed Juvenile Dermatomyositis (JDM) with 3/5 proximal muscle weakness. She did not have difficulty swallowing or evidence of lung disease. She had evidence of Gottron's papules without ulceration. She is ambulatory but demonstrates positive Gowers' sign. On laboratory evaluation, her muscle enzymes were elevated. She met criteria for moderate JDM and was started on CARRA CTP Protocol B with initiation of pulse IVMP x three days followed by weekly pulse steroids for two weeks, methotrexate 1mg/kg SQ once weekly (max 40mg) and prednisone 2mg/kg/day (max 60mg) once daily for four weeks, and IVIG 2g/kg q2 weeks x 3 sessions then monthly. Despite this therapy for four weeks, she has not shown significant clinical response. She was continued on this regimen for an additional 4 weeks with no change (CARRA CTP Protocol B – "Unchanged after four weeks" arm). After 8 weeks of therapy without significant clinical response, which of the following would you do next (either with or without additional pulse steroids)?
    a. No change
    b. ADD the following to the current regimen (you will have the opportunity to indicate what you would add/switch to on the next page)
    c. SWITCH Methotrexate to another therapy (you will have the opportunity to indicate what you would add/switch to on the next page)
    d. Other (please specify)
3. ADD the following to the current regimen.
    a. Add non-biologic DMARD at this time (more information to be collected on next page)
    b. Add biologic at this time
    c. Other (please specify)
4. Add non-biologic DMARD at this time.
    a. MMF
    b. Cyclosporine
    c. AZA
    d. Tacrolimus
    e. Cyclophosphamide
    f. Other non-biologic DMARD (please specify)
5. SWITCH Methotrexate to another therapy
    a. Switch to another non-biologic DMARD at this time (more information to be collected on next page)
    b. Switch to biologic at this time
    c. Other non-biologic DMARD (please specify)
6. Switch to another non-biologic DMARD at this time.
    a. MMF
    b. Cyclosporine
    c. AZA
    d. Tacrolimus
    e. Cyclophosphamide
    f. Other non-biologic DMARD (please specify)
7. After 12 weeks of therapy (after time of diagnosis) without significant clinical response, which of the following would you do next (either with or without additional pulse steroids)?
    a. No change

    b. ADD the following to the current regimen (more information to be collected on next page)
    c. SWITCH Methotrexate to another therapy (more information to be collected on next page)
    d. Other (please specify)
8. ADD the following to the current regimen.
    a. Add non-biologic DMARD at this time (more information to be collected on next page)
    b. Add biologic at this time
    c. Other (please specify)
9. Add non-biologic DMARD at this time.
    a. MMF
    b. Cyclosporine
    c. AZA
    d. Tacrolimus
    e. Cyclophosphamide
    f. Other non-biologic DMARD (please specify)
10. SWITCH Methotrexate to another therapy.
    a. Switch to another non-biologic DMARD at this time (more information to be collected on next page)
    b. Switch to biologic at this time
    c. Other (please specify)
11. Switch to another non-biologic DMARD at this time.
    a. MMF
    b. Cyclosporine
    c. AZA
    d. Tacrolimus
    e. Cyclophosphamide
    f. Other non-biologic DMARD (please specify)
12. If after 16 weeks (after time of diagnosis), the patient has been continued on DMARD therapy without significant improvement, during which time frame would you add biologic therapy?
    a. 16 weeks after diagnosis
    b. 20 weeks after diagnosis
    c. 24 weeks after diagnosis
    d. I would not start a biologic therapy
    e. Other (please specify)
13. How many DMARDs (alone or in combination) does the patient need to fail before you would start biologic therapy in this patient?
    a. One
    b. Two
    c. Three
    d. Four
    e. Other (please specify)
14. Which DMARDs does the patient need to fail before you would start biologic therapy in this patient (check all that apply)?
    a. Methotrexate
    b. MMF
    c. Cyclosporine
    d. AZA
    e. Tacrolimus

    f. Cyclophosphamide
    g. Other non-biologic DMARD (please specify)

15. Starting with your first choice, please rank the biologic therapies that you would use in this patient.
    a. Rituximab
    b. Abatacept
    c. Tocilizumab
    d. Infliximab
    e. Other
16. Case 1We should exclude anyone from the JDM Biologic CTP who has ever had any exposure to a previous biologic (excluding IVIG).
    a. Yes
    b. No
17. Case 2A JDM patient has previously received a biologic agent (other than Rituximab) prior to enrollment in the JDM biologic CTP. How long do they need to be off that biologic before enrolling into the JDM biologic CTP of a different arm?
    a. No restriction (ok to enroll right after)
    b. Washout for 4-5 half-lives or 1 month
    c. Other (please specify)
18. Case 3A JDM patient previously received Rituximab. How long afterwards can they be enrolled into the JDM biologic CTP of a different treatment arm?
    a. No restriction
    b. 6 months afterwards
    c. 6 months afterwards with detectable B cells
    d. Other (please specify)
19. If a JDM patient previously failed a TNF inhibitor >1 month or >4 half- lives ago, you should be able to enroll in the TNF inhibitor arm of the JDM biologic CTP?
    a. Yes, regardless of previous TNF inhibitor
    b. Yes – but only if tried a different TNF inhibitor
    c. No
20. If a JDM patient has been on a TNF inhibitor >1 month or >4 half-lives ago with previous benefit/clinical efficacy, you should be able to enroll in the TNF inhibitor arm of the JDM biologic CTP?
    a. Yes, regardless of previous TNF inhibitor
    b. Yes – but only if tried a different TNF inhibitor
    c. No
21. Case 5 a and bIf a JDM patient has previously failed abatacept >1 month or >4 half-lives ago, you should be able to enroll in the abatacept JDM Biologic CTP arm.
    a. Yes
    b. No
22. Case 5 a and beIf a JDM patient has previously been on abatacept >1 month or >4 half-lives ago with benefit/clinical efficacy, you should be able to enroll in the abatacept JDM Biologic CTP arm.
    a. Yes
    b. No
23. Case 6 a and bIf a JDM patient has previously failed tocilizumab 1 month or 4 half-lives ago, you should be able to enroll in the tocilizumab JDM Biologic CTP arm.
    a. Yes
    b. No

24. Case 6 a and bIf a JDM patient has previously been on tocilizumab 1 month or 4 half-lives ago with benefit/clinical efficacy, you should be able to enroll in the tocilizumab JDM Biologic CTP arm.
    a. Yes
    b. No
25. Case 7 a and bIf a JDM patient has previously failed Rituximab >6 months ago and now have detectable B cells, you should be able to enroll in the Rituximab JDM Biologic CTP arm.
    a. Yes
    b. No
26. Case 7 a and bIf a JDM patient has been on Rituximab >6 months ago and now have detectable B cells with previous benefit/clinical efficacy, you should be able to enroll in the Rituximab JDM Biologic CTP arm.
    a. Yes
    b. No
27. A JDM patient meets all criteria for a JDM biologic CTP arm and has just started that biologic therapy. Since they have already started that treatment and are continuing, we should exclude them from enrollment into the JDM biologic CTP for that medication arm
    a. Yes
    b. No
    c. Other (please specify)
28. Case 9For those that have started a biologic therapy consistent with a JDM biologic CTP arm, how long after starting that therapy would they be allowed to be enrolled?
    a. 1 week after starting the biologic
    b. 2 weeks after starting the biologic
    c. 1 month after starting the biologic
    d. Other (please specify)
29. A 7y/o female presented with predominately muscle weakness, diagnosed with JDM. She received induction therapy with: 3 doses of methylprednisolone 30mg/kg IV daily for 3 days followed by oral steroids 2mg/kg/day PO, methotrexate SQ 15mg/m2 weekly, hydroxychloroquine 5mg/kg PO daily and IVIG 2g/kg every 4 weeks, with methylprednisolone 30mg/kg monthly with each IVIG. Prednisone tapered to 0.5mg/kg/day over the course of 6 months with ongoing monthly IVIG and methylprednisolone pulse, but not able to further taper oral steroid dose due to flare of muscle disease. The decision was made to escalate treatment with addition of a biologic. In addition to starting a biologic, which of the following would you consider at the time of biologic initiation (choose 1):
    a. a. Continue current treatment of monthly methylprednisolone pulses
    b. b. Give “bridging” dose of methylprednisolone with 3 pulses daily for 3 days
    c. c. Give “bridging” dose of methylprednisolone with a pulse weekly for 4 weeks
    d. b and c
    e. a, b, and c
    f. Other (please specify)
30. A 7y/o female presented with predominately muscle weakness, diagnosed with JDM. She received induction therapy with: 3 doses of methylprednisolone 30mg/kg IV daily for 3 days followed by oral steroids 2mg/kg/day PO, methotrexate SQ 15mg/m2 weekly, hydroxychloroquine 5mg/kg PO daily and IVIG 2g/kg every 4 weeks. Prednisone tapered and discontinued at 6 months. Six months later, she developed mild weakness, increase in muscle enzymes, and new activity on nailfold capillaroscopy. A decision was made to start a biologic. In addition to starting a biologic, would you restart steroids at this time (choose 1)?

    a. Yes
    b. No
31. If you would restart steroids, which of the following regimens would you consider at the time of biologic initiation (choose 1).
    a. a. Give “bridging” dose of methylprednisolone with 3 pulses daily for 3 days
    b. b. Give “bridging” dose of methylprednisolone with a pulse weekly for 4 weeks
    c. a and b
    d. Give “bridging” dose of a single methylprednisolone pulse
    e. Restart oral steroids only
    f. Other (please specify)
32. For ongoing treatment plan, which of the following would you consider (choose 1).
    a. Daily oral steroids with taper
    b. Daily oral steroids with taper and monthly methylprednisolone pulse
33. You care for a 9-year-old female with difficult to control JDM. She has been maintained on prednisone, methotrexate, and hydroxychloroquine. Due to persistently active disease she was started on every 4 week IVIG (2 grams/kg) over a year ago. Unfortunately, over the past 2 months she has started to have a disease exacerbation consisting of worsening rash, weakness, and elevated muscle enzymes. Due to this, you have decided to start her on a biologic therapy. Should the patient be able to continue IVIG while starting a biologic therapy?
    a. Yes
    b. No
34. If yes, how long would you continue IVIG?
    a. Until they meet definition of clinical improvement
    b. At the discretion of the provider
    c. Other (please describe)
